# Soft-sediment deformation structures in Holocene coastal gravel deposits reveal two 1.8–2.0 ka old *M*_*w*_ > 7.0 earthquakes in southern-central Hispaniola

**DOI:** 10.1038/s41598-025-09922-y

**Published:** 2025-07-23

**Authors:** Francisco José Fernández, Fernando Pérez Valera, Javier Escuder-Viruete

**Affiliations:** 1 Departamento de Geología, Universidad de Oviedo, 33005 Oviedo, Spain; 2 Departamento de Ciencias de la Tierra y del Medio Ambiente, Universidad de Alicante, 03080 Alicante, Spain; 3 CSIC, Instituto Geológico y Minero de España, 28760 Madrid, Spain

**Keywords:** Natural hazards, Tectonics

## Abstract

An exceptionally well-preserved outcrop of Holocene liquefaction structures in the Tortuguero Beach of southern-central Hispaniola has been investigated. We present a new high-resolution orthoimage mosaic, combined with fieldwork, sedimentary logging, structural analyses, and rock sampling for granulometric, grain-shape, and geochronological analysis to improve our understanding of the seismic hazard and the magnitude of the cyclic paleo-earthquakes occurred in this high seismically active region. Our results revealed three sedimentary sequences of deformed layers separated by undeformed sections. These metric-scale, episodic liquefaction structures resulted in an unusual negative density gradient in a coarsening upward stratified succession. Deformed layers form NNW-trending dome and basin elongated structures controlled by the present-day NE-directed regional shortening. Radiocarbon dating of the lower, intermediate, and upper sequences yielded ages (1σ) of 2332—2008, 1982—1803, and 1770—1530 cal BP, respectively. Liquefaction structures were triggered by *M*_*w*_ > 7 earthquakes likely occurring every 200 years. Seismic hazard modeling establishes that the primary sources of earthquakes are the large-scale, strike-slip fault zones that accommodate the collision of the Beata Ridge with southern-central Hispaniola. These fault zones probably generated the 1751 *M*_*w*_ 7.5 Azua earthquake, and given the recurrence of such seismic events in southern Hispaniola, they could trigger future destructive earthquakes.

## Introduction

**Fig. 1:**
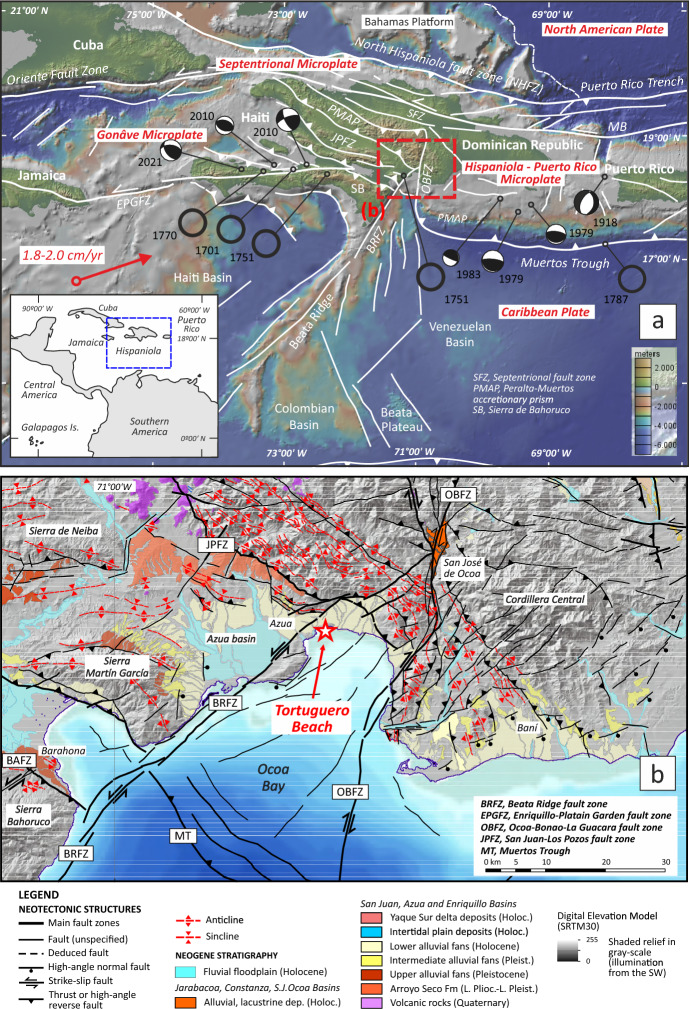
(**a**) Geodynamic context of the Hispaniola Island in the northern margin of the Caribbean Plate shows the microplate location and main fault zones. The red arrow defines the movement vector of the Caribbean Plate to the North American Plate (mod. Mann et al., 2002). Black circles, historical MI > 7.0 earthquakes until 1900 A.D.; beach balls, instrumental Mw > 5 earthquakes (also see Fig.  S4 in the supplementary note). (**b**) Neotectonic map of southern-central Hispaniola shows the Tortuguero Beach location in Ocoa Bay. Neotectonic structures and Quaternary lithostratigraphic units compiled in the map are from^4–6^. Topography in color scale and shaded relief in grayscale have been made from the GMRT synthesis data set^7^ with GeoMapApp (www.geomapapp.org).

The southern region of Hispaniola Island (Haiti and the Dominican Republic) has experienced 12 historical earthquakes with *M*_*w*_ > 6.5 (Fig. 1a; also see Fig. S4 in the supplementary note). Linking these earthquakes to specific faults is challenging due to the lack of reported surface ruptures. Instead, epicenter locations have been determined using damage reports and attenuation relationships^1,2^. An exception is the Enriquillo-Plantain Garden fault zone (EPGFZ), where strike-slip ruptures with a few meters of slip have been documented in southern Haiti and correlated with *M*_*w*_ 7.1–7.6 earthquakes^3^. A seismic quiescence period from the late eighteenth century to the 12 January 2010 *M*_*w*_ 7.0 Leogâne earthquake suggests a recurrence interval of about 240 years for the EPGFZ system^2^.

The soft-sediment deformation is frequent during earthquakes, and liquefaction-induced features have been used to illustrate the presence of seismic hazards and to deduce the location and magnitude of paleo-earthquakes^8–10^. Liquefaction occurs when loosely packed, water-logged sediments at or near the ground surface lose their strength in response to strong ground shaking. Gravel and sandy gravel sediments rarely undergo liquefaction due to the necessary rapid compaction. Liquefaction of these deposits is controlled by the variations of the gravel content and the relative density, and the case histories are recorded only during ground shaking generated by earthquakes *M*_*w*_ > 6^11^.

Soft-sediment deformation structures (SSDS), developed in Holocene gravel coastal sediments ranging in size from meters to tens of meters, were recently discovered at Tortuguero Beach in the Ocoa Bay of southern-central Hispaniola (Fig. 1b, also see Supplementary Video S1 online). Scale-intensity relationships also indicate that large earthquakes caused these liquefaction structures^12,13^, suggesting a high seismic hazard due to their proximity to the seismogenic source.

To better understand the occurrence and magnitude of large earthquakes in the region, we first analyze the geometry, sedimentary and structural features, spatial location, and age of the soft-sediment deformation structures found at Tortuguero Beach. Next, we correlate this paleo-earthquake record with historical seismic events, notably the destructive Azua earthquake of 18 October 1751, which affected Ocoa Bay^14^. Finally, we conduct a probabilistic seismic hazard analysis (PSHA) to identify fault zones linked to SSDS and assess seismic hazards in southern-central Hispaniola using our regional neotectonic knowledge^4–6^.

### Geological setting

The Peralta-Muertos accretionary prism forms from the NE-directed subduction of the Caribbean plate beneath the island-arc crust of Central Hispaniola, occurring from the middle Eocene to the late Oligocene^15–17^. From the early Miocene onward, it has recorded a history of collisions involving various crustal domains, particularly fragments of the Cretaceous Caribbean Large Igneous Province (CLIP). The oblique accretion of the northern CLIP resulted in the SW-directed Haitian-Neiba fold-and-thrust belt and the San Juan-Azua basins^16^. Since the Pliocene, the thickened crust of the central CLIP has caused the uplift of the Massif de la Selle-Sierra de Bahoruco and the formation of the Azua basin^15^. In the Quaternary, the collision of the Beata Ridge contributed to a recess in the Peralta-Muertos prism and the clockwise rotation of its structures^17^. The Beata Ridge fault zone (BRFZ) and the Ocoa-Bonao-La Guacara fault zone (OBFZ) mainly accommodate the strain produced by the Beata Ridge ongoing collision with southern-central Hispaniola, giving shape to Ocoa Bay^4–6^.

## Results

**Fig. 2:**
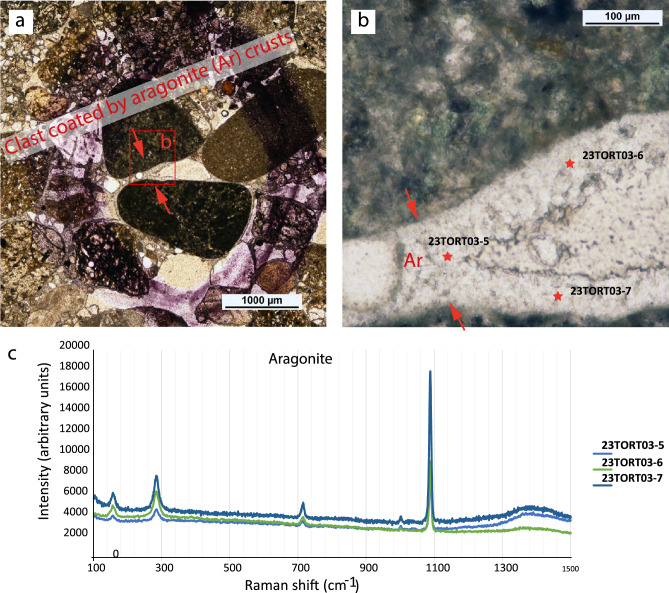
(**a**) Optical microscopy (OM) image of the cemented gravel deposit. (**b**) OM image details where the spots analyzed by RAMAN microscopy are located. (**c**) RAMAN diagram corresponding to aragonite spectra.

Tortuguero Beach, on its southwestern side, features a beachrock outcrop composed of coastal gravel and sand deposits that have undergone rapid cementation due to the precipitation of fibrous aragonite and high-magnesian types of calcite cement (Fig. 2). This rapid cementation increases the mechanical strength and stiffness of certains layers, producing semi-rigid horizons within the sedimentary succession. Regional tectonic uplift^6^, combined with marine wave action, have exposed part of this Holocene deposit. The beachrock is partially submerged during high tides and hurricanes.

What makes this beachrock outcrop particularly noteworthy is the subaerial exposition of 3 m-thick liquefied layers alternating with undeformed multilayer sequences of comparable thickness. This stratigraphy suggests three superimposed, diachronic records of liquefaction and rapid-cementation processes occurring within an area of high seismic activity. To our knowledge, such structures have not been documented in sandy gravel deposits. The rapid cementation following each earthquake event appears to be significantly influenced by the Caribbean climate, which favors fast carbonate precipitation. These cemented layers act as barriers to fluid flow during seismic shaking, promoting pore pressure buildup in adjacent less-cemented sediments and thereby facilitating liquefaction processes. This heterogeneous cementation is key to the development and preservation of SSDS, as it localizes deformation within specific layers and influences the geometry of deformation features such as dome-and-basin structures.

### Liquefaction structures in Tortuguero beach

The SSDS crop out in a 200 m-long and 25 m-wide beachrock outcrop, which dip seaward at approximately 10° (a large-scale image can be found as Supplementary Fig. S2 online). These deposits form a 6.85 m-thick succession of alternating centimeter-scale layers of gravel, sandy gravel and coarse-grained sand. The layers exhibit regular tabular bedding with low dip angles (6° and 14°) trending southward (i.e., seaward).

**Fig. 3:**
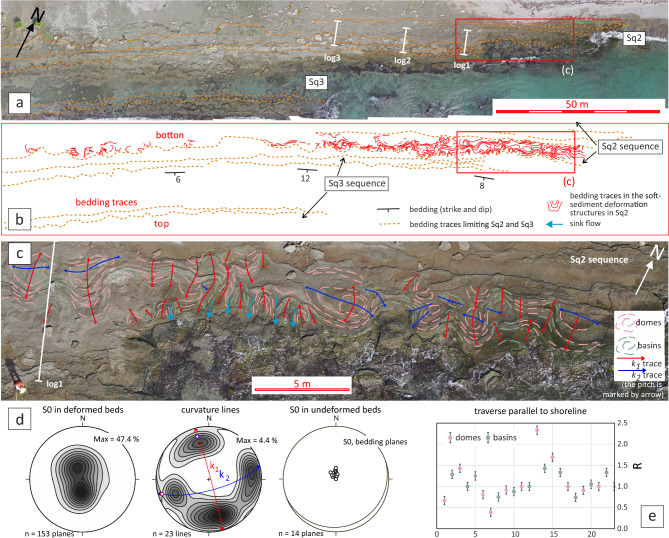
(**a**) The soft-sediment structures deforming the foreshore of Tortuguero Beach. The location of the stratigraphic logs included in Fig. 4 is indicated. (**b**) Scheme of relationships between stratigraphic sequences. Discontinuous and continuous red lines outline bedding traces, limiting sequences and deformed beds. Sq1 sequence is located northward, immediately out of the camera view. (**c**) Zoomed-in image of the liquefaction structures showing their internal structure in domes and basins. The two approximately orthogonal traces of the maxima curvature line *k*_*1*_ are shown by red (WNW-trending) and minimal curvature line by blue (ENE-trending) solid lines. (**d**) Contours in stereoplots at 0.5% intervals for the *S*_*0*_ poles in deformed beds, curvature lines measured in the dome and basin structures allowing to fit the mean orientation of* k*_*1*_ and *k*_*2*_, and *S*_*0*_ poles in undeformed beds. (**e**) the axial ratio R of the ellipses resulted from the intersection between the structures and the abrasion surface.

The succession comprises three sedimentary sequences, each several meters thick (Fig. 3). Each sequence consists of gravel and coarse sand layers that exhibit SSDS, separated by intervals of undeformed strata. From the base of the exposure (north) upwards (south), the sequence begins with a 0.75 m-thick deformed layers (Sq1), partially unconformably overlain by recent eolian sediments. Above this lies 1.20 m-thick set of deformed layers belonging to the intermediate sequence (Sq2), which in turn overlies a 1.10 m-thick package of undeformed beds from the same sequence. Finally, an upper liquefied sequence (Sq3) caps a 2.90 m-thick undeformed section of the same sequence.

**Fig. 4:**
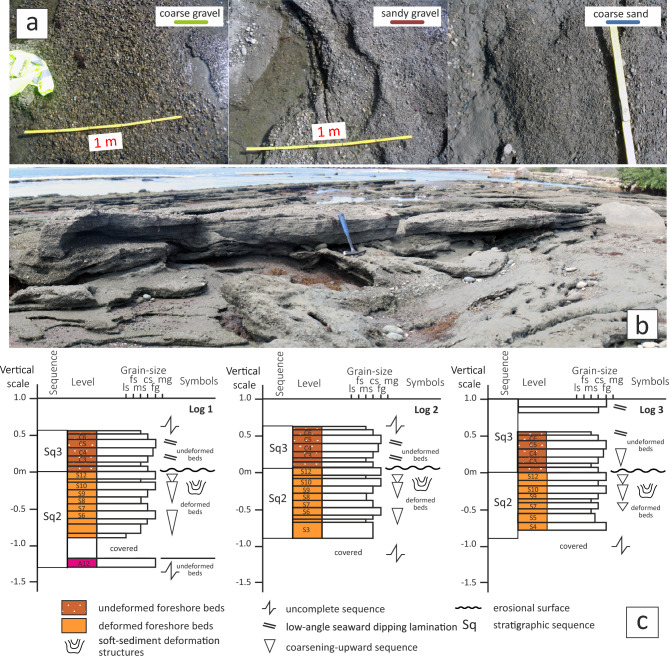
(**a**) Field aspects of the different lithologies comprise the foreshore deposits with soft-sediment deformation structures. (**b**) Detail the upper boundary of the middle Sq2 sequence with truncation by erosion of the beds below the base of the overlying Sq3 upper sequence (the view is towards the southwest and faces the sea). (**c**) Detailed stratigraphic logs of the middle Sq2 sequence that run parallel to each other (locations are shown in Fig. 3a). The deformed beds of the Sq2 sequence are unconformably overlain by the undeformed beds of the upper Sq3 sequence.

The intermediate sequence (Sq2) is exceptional well exposed, allowing observation of its complete internal structure. It consists of four upward coarsening sub-sequences, each beginning with coarse-grained sand and grading upwards into sandy gravel and gravel. The basal layer of Sq2 is 5–10 cm thick and consists of well-sorted coarse gravel. The second layer contains poorly sorted coarse sands mixed with gravel. The third consists of medium-grained sand with some silt laminae. The top of the Sq2 is marked by an erosional surface overlain by a flat, 10–25 cm-thick layer of coarse sand and gravel with inverse grading. The overlying Sq3 is 10–20 cm-thick and features undeformed beds with high-angle lamination typical of foreshore progradation facies. The other sequences show similar internal structures (Fig. 4), suggesting deposition in a high-energy, shallow marine coastal environment.

During sediment consolidation and pore-water expulsion, the bulk properties of the multilayer structure were strongly influenced by variations in grain-size and shape, as well as by later liquefaction^18,19^. Since liquefaction in gravels-rich sequences requires more energy than in finer grain-size sequences^11^ and is anomalous in upward-coarsening units^20^, we performed a detailed analysis of grain-size and shape variations in deformed layers of Sq2. Analyses were carried out on sections parallel to the bedding (Sec_XY) and on vertical sections perpendicular to bedding and aligned to the fluid scape conduits (Sec_XZ).

**Fig. 5:**
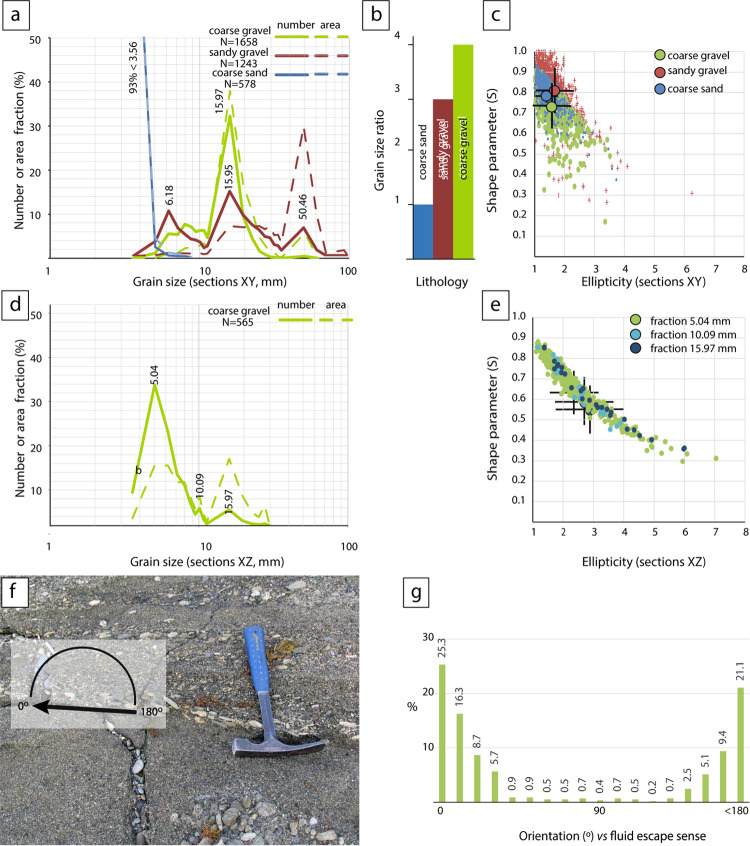
Grain size and shape analysis. (**a**) Representative grain size distribution of the sections XY normalized by number (solid line) or area fraction (dashed line). (**b**) The grain size ratio was determined according to the difference in grain size of the three types of lithological sorts. (**c**) Shape parameter (S) versus ellipticity of the grain distribution within the characterized lithological sorts and standard deviation of sections XY (see more details in Methods). (**d**) Normalized grain size distribution of section XZ is presented as a percentage of number (solid line) or area fraction (dashed line). (**e**) Shape parameter (S) versus ellipticity of the grain distribution within the coarse gravel sort and standard deviation for the fractions differentiated in section XZ. (**f**) The photograph displays lenticular clasts arranged in overlapping patterns along a fluid escape conduit. The arrow indicates the relative orientation of the grain to the fluid flow. 0° indicates the same direction as the fluid scape and 180° indicates the opposite sense. (**g**) The orientation histogram indicates well-defined imbrication in the lenticular gravel clasts, controlled by fluid escape.

In Sec_XY three-grain size sorts were identified: Coarse sand, with a unimodal distribution and 93% of particles finer than 3.56 mm; Coarse gravel, centered around 15.97 mm, comprising 32% of particle count and 38% of the area fraction; Sandy-gravel, with trimodal distribution showing peaks at 6.18, 15.95 and 50.46 mm (Fig. 5a). The coarse gravel sort are four times larger than the coarse sand sort (Fig. 5b). Despite the differences in size, all sorts show a similar ellipticity (< 2) and high roundness, with a shape parameter (*S*) ranging between 0.7–0.8 (Fig. 5c). In vertical Sec_XZ, grain-size tends to be finner than in Sec_XY (Fig. 5d), though the coarse fraction (< 20% area) still centers around 15.97 mm, matching Sec_XY mean.

Interestingly, in Sec_XZ, grain ellipticity increases as the *S* parameter decreases (Fig. 5e; see Supplementary Table S3 online), indicating that while roundness remains constant, particle shape transitions from spherical to lenticular. This transition is not observed in Sec_XY. Within fluid escape conduits, these lenticular clasts align with their long axis parallel to the direction of fluid movement (Figs. 5f,g). These variations in the grain-size and shape likely modified the permeability of gravel layers, increasing the viscosity contrast within the multilayer and making liquefaction possible once a critical threshold was reached^20^.

Fluid escape likely creates clast imbrication structures that reduce permeability in the coarse gravel layer and amplified in the viscosity contrast between the coarse and fine grain-size layers. Each sequence is truncated at the top and covered by an undeformed section composed of coarse-grained sand and gravel layers.

**Fig. 6:**
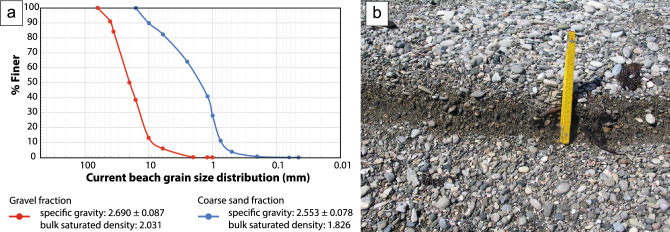
(**a**) Component scores for the coarser (gravel fraction, red curve) and finer (coarse sand fraction, blue curve) grains sampled from the current beach deposits. (**b**) Field aspects of the current foreshore sediments.

Because beachrock is lithified, it is used recent, unconsolidated beach sediments with similar grain-size distributions to measure specific gravity and bulk density under water-saturated conditions. Complementary sieve analyses of fine and coarse fractions yielded grain-size distributions consistent with those obtained from SSDS imagery (Fig. 6). Coarse sand and gravel exhibited specific gravity of 2.553 ± 0.078 and 2.690 ± 0.087 gr/cm^3^, respectively. When saturated, density decreased more in coarse sand than in gravel (Table 1), increasing the density contrast between the two.

**Table 1:** Specific gravity determination for current beach deposits using a water pycnometer (174 gr weight) procedure.

Sample code	Longitude (X)	Latitude (Y)	Sediments classification	M1 density (gr/cm^3^) and weight (gr)		M2 density (gr/cm^3^) and weight (gr)		M3 density (gr/cm^3^) and weight (gr)		M4 density (gr/cm^3^) and weight (gr)		Temperature water (°C)	Bulk saturated density (gr/cm^3^)	Specific gravity (gr/cm^3^)
23TORT02	− 70.6896	18.4279	Coarse sand	2.631	471	2.566	468	2.575	461	2.444	192	22.4	1826	2.553 ± 0.078
23TORT04	− 70.6896	18.4279	Gravel and cobble	2.636	461	2.644	476	2.792	201			22.4	2031	2.690 ± 0.087

M1, M2, M3, and M4 are the results of the tests in fractions of the sample with a specific weight that allows for determining a standard deviation for the specific gravity. Bulk saturated density is calculated using the whole weight of each sample.

**Fig. 7:**
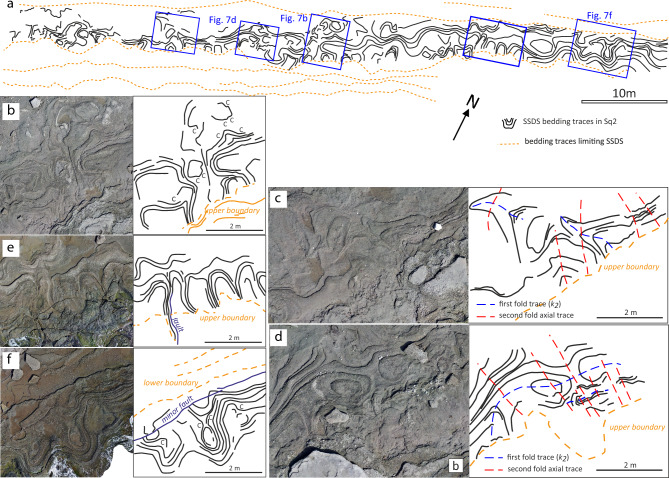
(**a**) Location of detailed orthophotos showing the interference geometry of SSDS in Sq2 view in a subhorizontal interpreted section XY. Orthoimages (left) and their interpretation (right): (**b**) 2-D folding interference pattern where coaptation (C) folds^21^ were found. (**c** and **d**) A second folding (dashed red line trace) modifies two-dimensional forms of interference patterns in asymmetric convoluted folds (blue dashed line trace). (**e**) The fault trace (blue solid lines) of a vertical fault lies parallel to the fluids scape conduit. (f) the fault trace of a vertical fault of centimetric separation lies parallel to the lower Sq2 boundary.

Structurally, SSDS are characterized by metre-scale convoluted folds that create a dome-and-basin form (Fig. 3). The interference between the beachrock abrasion surface and the convoluted folds produces ellipsoid shapes with low-aspect ratios and no preferred orientation. Pole diagram of these convoluted folds reveals two orthogonal directions: NNW-SSE (*k*_*1*_) and ENE-WSW (*k*_*2*_) corresponding to maxima and minima curvature lines^21^. These structures are truncated by the basal contact of overlying undeformed layers, indicating an erosional episode prior to their deposition (Fig. 7a).

**Fig. 8:**
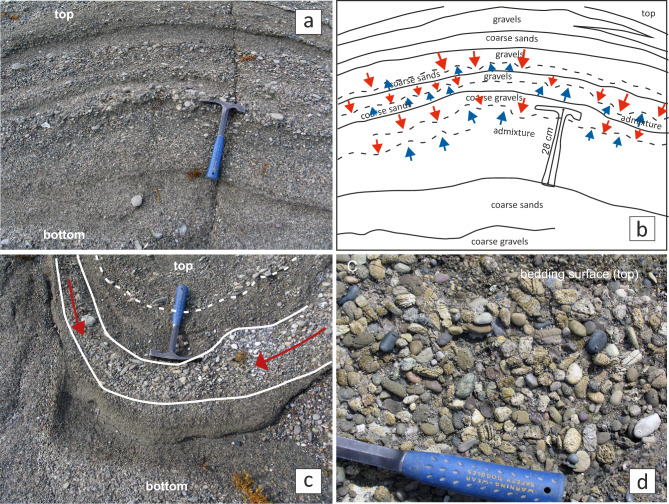
SSDS details observed in sections XZ: (**a**) the multilayer consists of sandy gravel, coarse sand and admixture layers consisting of bad-sorted gravelly sand but with grain size fractions similar to the well-sorted layers. (**b**) Interpretation of the stratigraphic succession, where gravitation collapses at the end of the liquefaction process, is induced by the load at the top of the denser gravel beds over the lower-density coarse sand bottom layers. (**c**) Due to gravitational segregation, the progressive gradation of gravel to finer pebbles and gravel in a layer from the basin hinges towards the limbs. (**d**) The appearance of most broken clasts with previous fractures and mechanical discontinuities was observed in section XY (parallel to the abrasion surface). However, the neighboring broken clasts are spatially related and some of the fractures are transgranular.

Closer inspection of the SSDS reveals complex fold interference patterns, including coaptation folds that accommodate spatial changes between folded layers^22^ (Fig. 7b), as well as interferences corresponding to second folds axial traces sub-parallel to *k*_*1*_, creating type-3 superposed fold patterns^23^ (Figs. 7c,d). Additionally, vertical faults with centimeter-scale apertures, trending parallel to *k*_*1*_ and *k*_*2*_ (Fig. 7e,f), further indicate that part of the deformation occurred under brittle conditions, requiring some degree of early cementation to sustain fracturing. Nonetheless, other liquefaction structures, such as the gravitational collapse of gravel beds, clast segregation and clasts breakage (Fig. 8), may also have formed in unconsolidated sediments^24^, highlighting the variability of mechanical conditions during deformation.

Together, these observations underscore the fundamental role of cementation heterogeneity in controlling deformation styles and structural complexity of the SSDS at Tortuguero Beach, with mineralogical cement types and distributions strongly influencing the fluid flow pathways, pre-pressure evolution, and the mechanical response to seismic loading.

#### Processes and magnitude

Based on the previous morphologies and a comparison with liquefaction forms observed in other study cases^8,25,26^, we interpret the SSDS at Tortuguero Beach as recurrent liquefaction structures. According to the case histories, these were likely produced by earthquakes *Mw* > 6^11^. In addition, the thickness and morphologies of SSDS studied also suggest *M*_*L*_ > 6^13^.

The main coseismic effects include gravel and sand boils that form dome and basin structures ranging from 1.5 to 3.0 m. in diameter, localized lateral spreading, and 5 to 20 cm of local subsidence. These phenomena notably impact the entire hectometre-scale outcrop area. According to the Environmental Seismic Intensity (ESI) scale^12^, these coseismic effects were generated by earthquakes with ESI between VIII and X. This further indicates that the Tortuguero Beach site was situated near the earthquake epicenter.

**Fig. 9:**
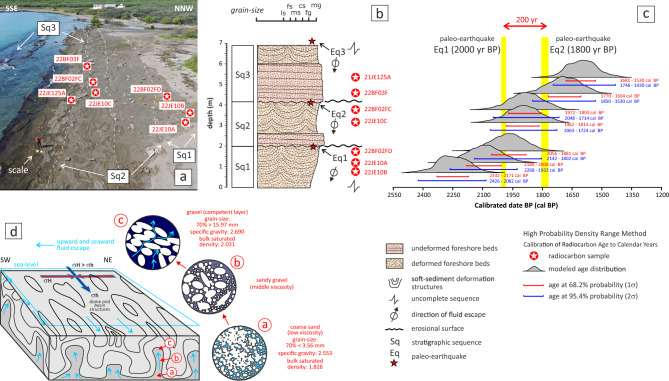
(**a**) Aerial view and (**b**) stratigraphic section of Tortuguero Beach showing the location of the stratigraphic sequences and geochronological samples. (**c**) Probability density plots (grey) denote age distributions for each sample modeled using the high-probability density range method. Radiocarbon ages are expressed as calibrated calendar intervals of years before present (BP) for a probability level of 95.4% (2σ, blue lines) and 68.2% (1σ, red lines). Age distributions for a probability level of 1σ allow us to constrain the age of two paleo-earthquakes at 2000 years BP (Eq. 1) and 1800 years BP (Eq. 2), separated by a recurrence interval of about 200 years. (**d**) Scheme of the density inversion in a water-saturated upward coarse-grained sequence that, during an earthquake, produces its gravitational instability, the mobilization of sands and their expulsion upwards, giving rise to SSDS.

During an earthquake, the fluid pressure increases according to forces generated by the seismic wave propagation. In these conditions, density inversion in a stratified sequence of gravels and sands produces a gravitational instability that conduits to liquefaction and the formation of SSDS. In each water-saturated coarsening upward sequence of Tortuguero Beach, the liquefaction of the sediments was controlled by a sand-gravel permeability higher than the threshold value of 3.5 times^20^ (Table 2), with an estimated permeability coefficient of 0.41 cm/s for sand and 1.99 cm/s for gravel. This permeability contrast value must be taken as a minimum if the effects of the differential package and compaction on the variations in roundness and ellipticity of the grain shapes during liquefaction are considered (Fig. 5). Additionally, the estimated bulk saturated density of the gravel beds (2.03 g/cm^3^) is slightly higher than that of the coarse-grained sand beds (1.82 g/cm^3^) (Table 1). Therefore, the contrast in permeability and the inversion in density could mobilize the well-sorted coarse sands underlying sandy gravels, then the ejection of fluids towards lower pore pressure sediment/rock volumes (Fig. 9).

**Table 2:**
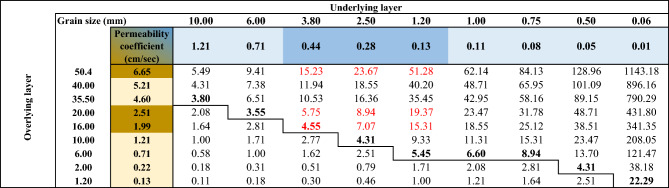
The matrix shows the permeability ratio between the sandy gravels of the overlying versus the underlying layers measured in the grain size fractions of the Tortugeros beachrock.

The ratios for which the SSDS might be formed^25^ are shown in bold, and the ratios of the respective more frequent fractions are remarked in red.

The homogeneous orientation of the lines of maximum curvature *k*_*1*_ and *k*_*2*_ in the dome and basin structures suggests that deformation is controlled by a combination of the local stress field and the likely lithological anisotropy axes of the affected volume. The type of SSDS observed at Tortugueros Beach, the size and orientation of the structures developed, as well as the grain size of the sequences liquefied, may be beneficial for validating or discharging the derived probabilistic hazard model for Ocoa Bay.

#### Radiocarbon ages and recurrence interval of paleo-earthquakes

Rare marine shell fragments of millimeter size are collected from the stratigraphic succession for radiocarbon dating. The obtained conventional radiocarbon ages were calibrated to calendar year equivalents using the high-probability density range method^27^, utilizing the Marine20 database^28^ (Table 3). Two bivalve fragments collected in the deformed beds of the intermediate Sq2 sequence have provided very similar modeled radiocarbon ages of 2048—1714 and 2063—1724 cal BP, for a probability level of 95.4% (2σ). Two other bivalve fragments from the undeformed beds of the overlying Sq3 sequence yielded modeled ages of 1746—1430 and 1850—1530 cal BP. Considering a probability level of 68.2% (1σ), the paleo-earthquake Eq. 2 took place approximately 1800 years ago (Fig. 9). Three bivalve fragments from the deformed beds of the lower Sq1 sequence yielded modeled radiocarbon ages of 2142—1802, 2268—1932 and 2426—2082 cal BP., for a probability level of 95.4%, partially overlapping with the age range of Sq2 sequence. Considering the maximum ages obtained in the Sq2 samples for a probability level of 1σ, together with the minimum ages modeled in two Sq1 samples, the age of the paleo-earthquake Eq. 1 can be tentatively assumed at 2000 years BP (Fig. 9). The two suggested earthquakes occurred into an interval of about 200 years.

**Table 3:** Radiocarbon dates are presented as conventional radiocarbon and uncalibrated measured AMS ^14^C ages, with information on the dated material and its geographical location.

Sample code	Longitude (X)	Latitude (Y)	Relative stratigraphic depth (cm)	Measured AMS ^14^C age in BP^a^	Error (year)	Calibration curve	Delta R (year)	Delta R Error (year)	Conventional radiocarbon age (BP)	Calibrated age in BP^b^ (2σ)	Calibrated age in BP^b^ (1σ)	Significance for event chronology
21JE125A	− 70.6891	18.4281	532	1600	± 30 BP	MARINE20	− 161	± 24	2030 ± 30 BP	1746–1430	1681–1530	pre−E3
22BF03F	− 70.6897	18.4279	458	1690	± 30 BP	MARINE20	− 161	± 24	2100 ± 30 BP	1850–1530	1770–1604	pre−E3
22BF02FC	− 70.6895	18.4281	376	1850	± 30 BP	MARINE20	− 161	± 24	2260 ± 30 BP	2048–1714	1972–1803	pre−E2
22JE10C	− 70.6896	18.4280	312	1860	± 30 BP	MARINE20	− 161	± 24	2270 ± 30 BP	2063–1724	1982–1814	pre−E2
22BF02FD	− 70.6895	18.4281	174	1900	± 30 BP	MARINE20	− 161	± 24	2330 ± 30 BP	2142–1802	2056–1881	pre−E1
22JE10A	− 70.6896	18.4279	118	1890	± 30 BP	MARINE20	− 161	± 24	2330 ± 30 BP	2268–1932	2180–2008	pre−E1
22JE10B	− 70.6896	18.4279	78	2150	± 30 BP	MARINE20	− 161	± 24	2560 ± 30 BP	2426–2082	2332–2171	pre−E1

Quality assurance measurements												
*Reference material 1*												
Expected value	0.44 ± 0.04 pMC											
Measured value	0.44 ± 0.04 pMC											
Agreement	Accepted											
*Reference material 2*												
Expected value	129.41 ± 0.06 pMC											
Measured value	129.43 ± 0.39 pMC											
Agreement	Accepted											
*Reference material 3*												
Expected value	96.69 ± 0.50 pMC											
Measured value	96.89 ± 0.30 pMC											
Agreement	Accepted											

Known-value reference materials were analyzed quasi-simultaneously with the unknowns.

Results are reported as expected values versus measured values.

Reported values are calculated relative to NIST SRM-4990C and corrected for isotopic fractionation.

Results are reported using the direct analytical measure percent modern carbon (pMC) with one relative standard deviation.

Agreement between expected and measured values is taken as being within 2σ agreement (error × 2) to account for total laboratory error.

Dated material are shells of marine bivalve mollusks. Radiocarbon dating was performed at the beta analytic laboratory.

^a^Conventional radiocarbon age in years before present (BP), where “present” is AD 1950, without δ ^13^C correction.

^b^Calibrated ages have been calculated using the Radiocarbon Calibration Program BetaCal4.20 and the Marine20 calibration curve, with Delta-R = − 161 ± 24 derived for marine samples^29^. Age ranges are given at 2σ (95.4%) probability level. The reported results are accredited to ISO/IEC 17,025:2017 Testing Accreditation PJLA #59,423 standards.

## Discussion

The historical seismic record for south-central Hispaniola spans approximately the past 525 years. Epicenter locations for pre-instrumental events are inferred from reports of earthquake damage and regional attenuation relationships between epicentral intensity and distance^1,2^. For instance, the exact location and magnitude of the 1684 (*M*_*w*_ 6.0, which destroyed Azua) and 1691 (*M*_*w*_ 7.5, which destroyed Santo Domingo) earthquakes remain uncertain, as no associated rupture surfaces have been identified. Similarly, for the 18 October, 1751 *M*_*w*_ 7.5 Azua earthquake (Fig. 1a), both Peralta-Muertos accretionary prism and the eastern end of the EPGFZ have been proposed as potential seismogenic sources^2^. The same uncertainty applies to other historical earthquakes, including those of 1615, 1684, 1691, and 1911, with estimated magnitudes ranging from* M*_*w*_ 6.0 to 7.5 (also see Supplementary Note online). Consequently, due to limited knowledge of their macroseismic effects, confidently associating these historical earthquakes with specific fault zones remains problematic, complicating seismic hazard assessment.

From a hazard perspective, a key question is whether seismic events of similar magnitude to those that formed the SSDS at Playa Tortuguero are likely to recur. Addressing this question requires identification of potential seismogenic sources and modeling the seismic hazard in the region. Probabilistic seismic hazard analysis (PSHA) provides a framework for quantifying the likelihood of ground shaking at or above a given intensity at a specific location by combining models of earthquake occurrence and ground motion^30,31^. PSHA-derived maps typically express ground motion in terms of peak ground acceleration (PGA) for a defined return period or exceedance probability^32^.

Recent PSHA studies in south-central Hispaniola^5,6^ have incorporated the spatial distribution of the major seismogenic structures, including the San Juan-Pozos Fault Zone (JPFZ), Ocoa-Bonao-La Guacara Fault Zone (OBFZ), Beata Ridge Fault Zone (BRFZ), Bahoruco Fault Zone (BAFZ), Muertos Trough (MT) and the eastern tip of the EPGFZ (Fig. 1; see also Supplementary Table S4 online).

**Fig. 10:**
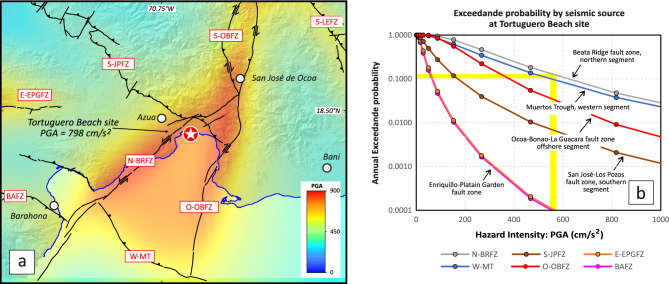
(**a**) Result of the probabilistic seismic hazard modeling in southern-central Hispaniola, expressed as iso-PGA (Peak Ground Acceleration) zones (in cm/s^2^) for a return period of 475 years (i.e., an exceedance probability of 10% in 50 years). The figure also includes the trace of the main seismotectonic structures described in the Supplementary Material. Shaded relief and abbreviations as in Fig. 1. The high modeled PGA value of 798 cm/s^2^ at Tortuguero Beach is consistent with the formation of the observed liquefaction structures by a strong ground shaking during a nearby Mw > 6.0 earthquake. (**b**) Disaggregation of the hazard by seismic source suggests that ruptures along the N-BRFZ, the W-MT, and less likely, the O-OBFZ segments could have generated PGA values > 600 cm/s^2^ at Tortuguero Beach, triggering the formation of the observed liquefaction structures. Shaded relief in grayscale has been made from the GMRT synthesis data set^7^ with GeoMapApp (www.geomapapp.org).

These analyses identify a zone of elevated seismic hazard around Ocoa Bay, where PGA values > 500 cm/s^2^ define a triangular zone associated with the BRFZ, OBFZ, and MT (Fig. 10a), with minimal contributions from the other fault systems. Considering that *M*_*w*_ > 7.0 earthquakes can produce PGA values between 680 and 1660 cm/s^2^ at distances of 1–10 km in poorly consolidated sediments^32^, the modeled PGA of 798 cm/s^2^ at Tortuguero Beach suggests a proximity to the source. This value also falls within the range of ground shaking capable of inducing gravel liquefaction^11,33^.

Hazard disaggregation by a seismic source indicates that ruptures along the N-BRFZ (12% contribution), the W-MT (10%) and, to a lesser extent, the O-OBFZ (4%) are capable of producing PGA values > 550–600 cm/s^2^ at Tortuguero Beach (Fig. 10b), at a 10% exceedance probability. The presence of the SSDS at Tortuguero Beach, formed under seismic shaking consistent with PGA values predicted by the PSHA model, provides tangible geological evidence supporting the validity of these hazard assessments.

Finally, the areal hazard distribution inferred from PSHA modeling also supports the hypothesis that either the BRFZ or the MT may have generated the 1751 *M*_*w*_ 7.5 earthquake, which destroyed the nearby city of Azua, according to some historical chronicles, produced a tsunami in Ocoa Bay. Thus, the SSDS serves as a crucial proxy for past large-magnitude earthquakes, underscoring the potential for similar future seismic events and reinforcing the importance of incorporating geological data into seismic hazard evaluations and regional risk mitigation strategies.

## Conclusions

The soft-sediment deformation structures observed in gravel coastal deposits at Tortuguero Beach represent episodic and exceptionally well-preserved liquefaction structures. They form as a result of a negative density gradient within an upward-coarsening stratigraphic sequence. The deformed multilayer exhibits dome-and-basin structures controlled by the present-day NE-directed regional shortening. Large earthquakes (*M*_*w*_ > 7) have produced these SSDS, which are estimated to occur roughly every 200 years. These events are likely triggered by major fault zones that accommodate the active Beata Ridge collision to southern-central Hispaniola. These faults probably generated the 1751 *M*_*w*_ 7.5 Azua earthquake and, considering the seismic cycle duration, could be the source of a future destructive earthquake.

### Methods

The tectonic interpretation was based on compiling high-resolution orthoimages obtained using an uncrewed aerial vehicle (UAV) with 10 cm resolution.

X-ray diffraction was employed to determine the mineralogical composition of carbonate-cement in the coated gravels of the beachrock. Analyses were conducted using a confocal microscopy Olympus BXFM-ILHS equipped with a Raman dispersive Jobin Yvon labRam HR UV 800 spectroscope (Ar 532 nm laser and 785 nm diodes) with a CCD detector.

Grain-size analyses involved photographing differentiated lithologies parallel to the top bed (sections XY) and vertical sections (sections XZ) orthogonal to the bed and parallel to the fluid escape structures. Grain-size and shape analyses were performed on XY sections of the three differentiated sorts; however, only suitable XZ sections of the coarse gravel sort were obtained. These photographs were used to map areas larger than 2 m^2^, outlining the grain size. The shape parameter *S*^29^ is calculated as

1 $$S = \frac{4\pi A}{{P^{2} }}$$

After measuring the area (*A*) and perimeter (*P*) of grain in pixels, all grains smaller than 20 pixels^2^ were excluded. The value of *S*, which represents the degree of irregularity in the shape of the grain, ranges between 0 and 1. A value of 1 indicates a perfectly circular grain, while 0 indicates increasing irregularities in the grain shape. It should be noted that S is not dependent on the size of the grain. Grain-size was measured as the radii equivalent (*r*_*i*_) of each grain, were

2 $$r_{i} = \sqrt {\frac{{\left( {A + P} \right)}}{\pi }}$$

Complementary sieve analyses of the finer and coarser sorts in samples collected from the current beach deposits that overlay the beachrock have been carried out using a pycnometer of 350 ml to measure the specific gravity and saturated bulk density in four coarse sand tests and three coarser gravel tests.

Marine shell samples of the coastal deposits were collected for radiocarbon dating. Fossils were initially examined using a scanning electron microscope (SEM) and analyzed by X-ray diffraction (XRD) to assess recrystallization, given that aragonitic shells should not contain calcite unless recrystallized. Samples were then prepared, bleached, and analyzed using four NEC accelerator mass spectrometers (AMS) and four Thermo IRMS instruments in Beta Analytic Testing Laboratory (www.betalabservices.com). Radiocarbon ages were reported as years before present (BP, where “present” = AD 1950), rounded to the nearest 10 years. Ages exceeding the modern reference are expressed as percent modern carbon (pMC), with the modern standard being 95% of the 14C signature of NIST SRM-4990C (oxalic acid). Quoted errors are 1σ counting statistics. Calibration to calendar year was performed using BetaCal4.20^27^ and the MARINE20 database^28^, applying a reservoir correction (ΔR = − 161 ± 24), as calculated from the Beta website (http://calib.org/marine/; last access: 28 February 2025) based on the local values^29^. Quasi-simultaneous analysis of reference materials with the samples was used to validate the radiocarbon analyses. Quality assurance results are reported as expected values versus measured values, which were calculated relative to NIST SRM-4990C and corrected for isotopic fractionation. Results are reported using the direct analytical measure of percent modern carbon (pMC) with one relative standard deviation. Expected and measured values are taken as being within 2σ agreements to account for total laboratory error.

Seismic hazard in southern-central Hispaniola was estimated through a regional-scale probabilistic seismic hazard analysis (PSHA)^32^, utilizing a recent seismotectonic model of active fault zones^5,6^. The main seismotectonic structures are detailed in Supplementary Table S4. Hazard maps present ground shaking levels as peak ground acceleration (PGA, in cm/s^2^), for 475 years return period (10% exceedance probability in 50 years)^32^. PSHA employed the R-CRISIS code^34,35^, which incorporated spatial distribution of seismogenic sources, the occurrence and magnitude, and the ground motion attenuation. Maximum rupture magnitude per fault, or segment fault, was estimated from historical records and scaling relations ^36^, accounting for uncertainties^37^. Displacement rates were derived from geological data and/or geodetic GPS data^5,6,38,39^. The model considers four seismic source types 11, 12, 13: subduction, reverse, and strike-slip fault zones, concentrated large seismicity, and intervening areas of moderate to low seismicity. PSHA was conducted on a rectangular point grid spanning 17.960° N–18.835° N latitude and − 70.175° W to − 71.250° W longitude (WGS84), with 0.025° spacing. Results were interpolated using an inverse distance-squared weighting algorithm. Ground-shaking intensity for rock-ground site conditions in southern-central Hispaniola is shown in Fig. 10. Two modeling approaches were applied: (a) combined seismic sources and (b) individual seismic sources (see also Fig. S5 included in Supplementary Note online).

## Supplementary Information

Supplementary Information 1.

Supplementary Information 2.

Supplementary Information 3.

Supplementary Information 4.

Supplementary Information 5.

## Data Availability

All data generated or analysed during this study are included in this published article [and its supplementary information files].

## References

[1] ten Brink, U. S., Bakun, W. H. & Flores, C. H. Historical perspective on seismic hazard to Hispaniola and the northeast Caribbean region. *J. Geophys. Res. Solid Earth.* **116**, B12318 (2011).

[2] Bakun, W. H., Flores, C. H. & Uri, S. Significant earthquakes on the Enriquillo fault system, Hispaniola, 1500–2010: Implications for seismic hazard. *Bull. Seismol. Soc. Am.* **102**, 18–30 (2012).

[3] Prentice, C. S. et al. Seismic hazard of the Enriquillo-Plantain Garden fault in Haiti inferred from palaeoseismology. *Nat. Geosci.* **3**(11), 789–793 (2010).

[4] Escuder-Viruete, J., Fernández, F. J., Valera, F. P. & Medialdea, A. Southern central hispaniola: a transition zone between oceanic subduction and arc-oceanic plateau collision. *Tectonics.* **42**(4), e2022TC007618 (2023).

[5] Escuder-Viruete, J., Fernández, F. J., Pérez Valera, F. & McDermott, F. Active tectonics, quaternary stress regime evolution and seismotectonic faults in southern central Hispaniola: implications for the quantitative seismic hazard assessment. *Geochem. Geophys. Geosyst.* **25**, e2023GC011003 (2024).

[6] Escuder-Viruete, J., Fernández, F. J., Valera, F. P., Medialdea, A. & Castillo-Carrión, M. Present-day shortening accommodated by folding, thrusting and strike-slip faulting in the enriquillo basin of southern central Hispaniola: implications for the regional seismic hazard. *Tectonics.* **44**(1), e2024TC008376 (2025).

[7] Ryan, W. B. F. et al. Global multi-resolution topography (GMRT) synthesis data set. *Geochem. Geophys. Geosyst.* **10**, Q03014 (2009).

[8] Obermeier, S. F. Use of liquefaction-induced features for paleoseismic analysis—an overview of how seismic liquefaction features can be distinguished from other features and how their regional distribution and properties of source sediment can be used to infer the location and strength of Holocene paleo-earthquakes. *Eng. Geol.* **44**(1–4), 1–76 (1996).

[9] Obermeier, S. F., Olson, S. M. & Green, R. A. Field occurrences of liquefaction-induced features: A primer for engineering geologic analysis of paleoseismic shaking. *Eng. Geol.* **76**(3–4), 209–234 (2005).

[10] Owen, G. & Moretti, M. Identifying triggers for liquefaction-induced soft sediment deformation in sands. *Sediment. Geol.* **235**, 141–147 (2011).

[11] Pokhrel, A., Chiaro, G., Kiyota, T. & Cubrinovski, M. Liquefaction characteristics of sand-gravel mixtures: Experimental observations and its assessment based on intergranular state concept. *Soils Found.* **64**(2), 101444 (2024).

[12] Michetti, A. M., et al. Intensity scale ESI 2007, in *Mem. Descr. Carta Geologica d’Italia, Servizio Geologico d’Italia, Dipartimento Difesa del Suolo* (eds. Guerrieri, L., & Vittori, E.) vol. 74, (APAT, 2007).

[13] Berra, F. & Felletti, F. Syndepositional tectonics recorded by soft-sediment deformation and liquefaction structures (continental Lower Permian sediments, Southern Alps, Northern Italy): Stratigraphic significance. *Sediment. Geol.* **235**(3–4), 249–263 (2011).

[14] Flores, C. F., ten Brink, U. S. & Bakun, W.H. Accounts of damage from historical earthquakes in the Northeastern Caribbean to aid in the determination of their location and intensity magnitudes. in *Open-File Report 2011–1133*, 237 (U.S. Geological Survey, 2012).

[15] Mann, P., McLaughlin, P. P. & Cooper, J. C. Geology of the Enriquillo-Azua basins, Dominican Republic, 2. Structure and tectonics, in *Geologic and Tectonic Development of the North America-Caribbean Plate Boundary in Hispaniola* (ed. Mann, P.) Spec. Pap. 262, 367–389 (Geol. Soc. Am., 1991).

[16] Heubeck, C. & Mann, P. Structural geology and Cenozoic tectonic history of the southeastern termination of the Cordillera Central, Dominican Republic, in *Geologic and Tectonic Development of the North America-Caribbean Plate Boundary in Hispaniola* (Ed. Mann, P.) Spec. Pap. 262, 315–336 (Geol. Soc. Am., 1991).

[17] Hernáiz-Huerta, P. P. & Pérez-Estaún, A. Estructura del cinturón de pliegues y cabalgamientos de Peralta. *República Dominicana. Acta geol. hisp.* **37**, 183–205 (2002).

[18] Lowe, D. R. Water escape structures in coarse-grained sediments. *Sedimentology* **22**(2), 157–204 (1975).

[19] Allen, J. R. L. The possible mechanics of convolute lamination in graded sand beds. *J. Geol. Soc.* **134**(1), 19–31 (1977).

[20] Dasgupta, P. & Chatterjee, A. Formation of water-escape structure during shock-induced fluidization: The role of permeability contrast. *J. Struct. Geol.* **124**, 1–7 (2019).

[21] Lisle, R. J. & Toimil, N. C. Defining folds on three-dimensional surfaces. *Geology* **35**(6), 519–522 (2007).

[22] Stauffer, M. Fold interference structures and coaptation folds. *Tectonophysics* **149**(3–4), 339–343 (1988).

[23] Ramsay, J. G. Interference patterns produced by the superposition of folds of similar type. *J. Geol.* **70**(4), 466–481 (1962).

[24] Morsilli, M., Giona Bucci, M., Gliozzi, E., Lisco, S. & Moretti, M. Sedimentary features influencing the occurrence and spatial variability of seismites (late Messinian, Gargano Promontory, southern Italy). *Sediment. Geol.* **401**, 105628 (2020).

[25] Idriss, I. M. & Boulanger, R. W. Soil liquefaction during earthquakes. Earthquake Engineering Research Institute, 237. (USA, 2008).

[26] Caputo, R. et al. Palaeoseismological evidence for the 1570 Ferrara earthquake. *Italy. Tectonics* **35**(6), 1423–1445 (2016).

[27] Ramsey, B. C. Bayesian analysis of radiocarbon dates. *Radiocarbon* **51**, 337–360 (2009).

[28] Heaton, T. et al. Marine20—the marine radiocarbon age calibration curve (0–55,000 cal B.P.). *Radiocarbon* **62**(4), 779–820 (2020).

[29] Fernández, F. J., Menéndez-Duarte, R. Aller, J. & Bastida, F. Application of geographical information systems to shape-fabric analysis, in *High-Strain Zones: Structure and Physical Properties*, (eds. Bruhn, D. & Burlini, L.). Spec. Publ., 245, 409–420 (Geol. Soc. Lond., 2005).

[30] McGuire, R. K. Seismic hazard and risk analysis. Earthquake Engineering Research Institute, 221 (USA, 2004).

[31] Morell, K. D. et al. Seismic hazard analyses from geologic and geomorphic data: Current and future challenges. *Tectonics* **39**, e2018TC005365 (2020).

[32] Gerstenberger, M. C. et al. Probabilistic seismic hazard analysis at regional and national scales: state of the art and future challenges. *Rev. Geophys.* **58**, e2019RG000653 (2020).

[33] Zhou, Y., Xia, P., Ling, D. & Chen, Y. Liquefaction case studies of gravelly soils during the 2008 Wenchuan earthquake. *Eng. Geol.* **274**, 105691 (2020). 10.1016/j.dib.2020.106308 PMC7522089 33015255

[34] Ordaz, M., Cardona O., Salgado-Gálvez M. A., Bernal G., Singh K. & Zuloaga D. Probabilistic seismic hazard assessment at global level. *Int. J. Disaster Risk Reduct.*, **10**(B), 419–427 (2014).

[35] Ordaz, M., & Salgado-Gálvez, M.A. R-CRISIS v20: Validation and Verification Document, *ERN Technical Report*, 308 p. (Mexico City, 2020).

[36] Stirling, M., Goded, T., Berryman, K. & Litchfield, N. Selection of earthquake scaling relationships for seismic-hazard analysis. *Bull. Seismol. Soc. Am.* **103**(6), 2993–3011 (2013).

[37] Bertil, D., Terrier, M. & Belvaux, M. Análisis de las fuentes sísmicas y evaluación de la amenaza sísmica regional del gran Santo Domingo. Estudio de la amenaza sísmica y vulnerabilidad física del Gran Santo Domingo. Informe Actividad, *BRGM/RP-65305-FR*. 149. (BRGM France, 2015).

[38] Calais, E., Symithe, S., Mercier de Lépinay, B. M. & Prépetit, C. Plate boundary segmentation in the northeastern Caribbean from geodetic measurements and Neogene geological observations. *C. R. Geósci.* **348**, 42–51 (2016).

[39] Mann, P. et al. Oblique collision in the northeastern Caribbean from GPS measurements and geological observations. *Tectonics* **21**(6), 1–23 (2002).

