# Supplementary figures and images for: Soft-sediment deformation structures in Holocene coastal gravel deposits reveal two 1.8–2.0 ka old Mw > 7.0 earthquakes in southern-central Hispaniola
Source: Sci Rep. 2025 Jul 23;15:26793. doi: 10.1038/s41598-025-09922-y (PMC12287264; doi:10.1038/s41598-025-09922-y)

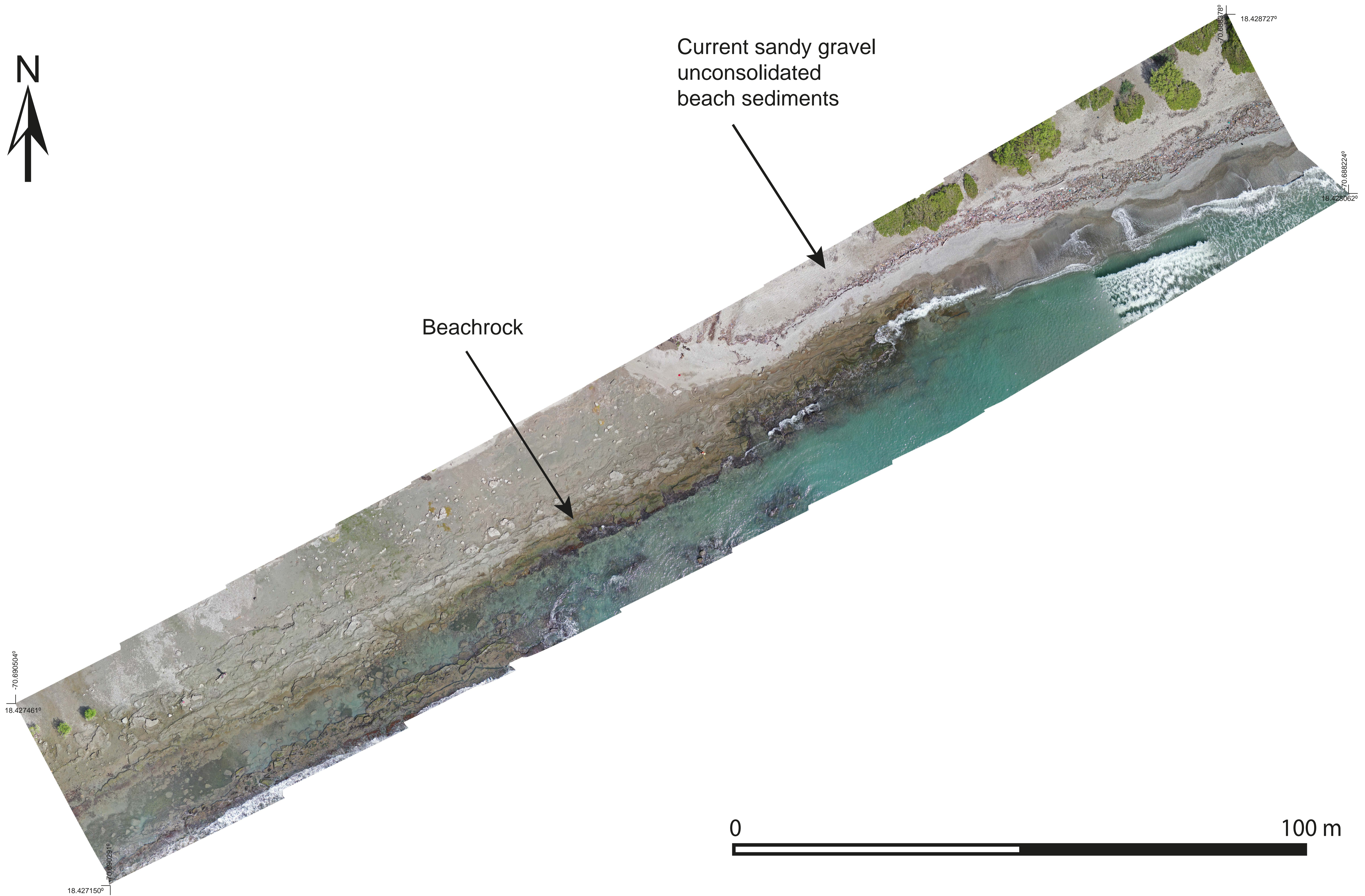

Figure S2: A high-resolution version of the general orthoimage compiled for Tortuguero Beach

Supplement: Supplementary file 1 — Supplementary Information 1. [file 41598_2025_9922_MOESM1_ESM.pdf]
