# Supplementary material for: Soft-sediment deformation structures in Holocene coastal gravel deposits reveal two 1.8–2.0 ka old Mw > 7.0 earthquakes in southern-central Hispaniola
Source: Sci Rep. 2025 Jul 23;15:26793. doi: 10.1038/s41598-025-09922-y (PMC12287264; doi:10.1038/s41598-025-09922-y)
Supplement: Supplementary file 5 — Supplementary Information 5. [file 41598_2025_9922_MOESM5_ESM.docx]

***Soft-sediment deformation structures in Holocene coastal gravel deposits reveal two 1.8-2.0 ka old M_w_ > 7.0 earthquakes in southern-central Hispaniola,***

**Supplementary Note**

Francisco José Fernández^1*^, Fernando Pérez-Valera^2^, and Javier Escuder-Viruete^3^

*^1^Universidad de Oviedo, Departamento de Geología, 33005, Oviedo, Spain.*

*^2^ Universidad de Alicante, Departamento de Ciencias de la Tierra y del Medio Ambiente, 03080, Alicante, Spain.*

*^3^Instituto Geológico y Minero de España-CSIC, Madrid, Spain.*

***** *fjfernandez@uniovi.es*

**Contents**

1. Records of historical earthquakes

2. Seismic hazard assessment in southern-central Hispaniola

3. Ethics statement

4. References

**1. Records of historical earthquakes**

This section is derived from the USGS open-file report 2011-1133 ^1^, which provides descriptions of damages caused by earthquakes that occurred in the northeast Caribbean region over the past 500 years. The report used various historical documents, including colonial catalogs, letters, books, and the general Archive of the Indies of 1785 (Portal de Archivos Españoles; <http://pares.mcu.es/>). Fig. S4 shows the location of main historical earthquakes (*M_W_* > 7.0) until 1900 AD and instrumental earthquakes (*M_W_* > 5).

^2, 3, 4^ used the descriptions of damage caused by historical seismic events to assign intensities and derive regional attenuation relations. These authors analyzed two sets of intensity assignments (*M_I_*) to assess the uncertainty introduced by their subjective assignment. They found an intensity attenuation relation similar to that obtained for southern California ^5^, which is characterized by a faster attenuation than that obtained for earthquakes in Puerto Rico or the stable continental region of eastern North America. The first recorded earthquake in Hispaniola was the 2 December 1562 *M_w_* 7.7 ^1, 2^. These authors suggest that this earthquake may have been triggered by the Septentrional fault zone (SFZ) rupture in northern Hispaniola. The 7 May 1842 *M_w_* 7.6 earthquake was also probably produced by the activity of the SFZ. The recurrence interval for earthquakes in northern Hispaniola is around 240 years ^2^, similar to the recurrence interval for the Enriquillo-Platain Garden fault zone (EPGFZ) system and the western segment of the Muertos Trough. Therefore, Hispaniola's northern and southern regions have a similar recurrence interval for large earthquakes of about 240 years.

Although there is no record of large earthquakes in the Muertos Trough, five of the most significant earthquakes (with *M_W_* between 6.8 and 7.5) that affected southern Hispaniola during the 17th century are tentatively situated in the forearc of the Muertos accretionary prism (Fig. S4). The Tortuguero Beach site could be located within the attenuation area of these large earthquakes. The location for the epicenter of the 18 October 1751 *M_I_* 7.4 - 7.5 destructive earthquake is proposed near the Tortugeros Beach site ^2^. For these authors, it could be located either offshore westward of the *M_s_* 6.7 thrust event that occurred on the Los Muertos Trough on 24 June 1984 ^6^ or onshore eastward of the old location of the Azua city (now called Pueblo Viejo), which was completely destroyed by the earthquake. Other significant damaged areas include Santo Domingo, Cotuí, Hinche and La Vega. Although the intensity centers and *M_I_* depend on the intensity assigned for Santo Domingo city, an onshore epicenter is proposed as the most likely one ^2^. In favor of this assignment are the sulfur springs that appeared in the mountains north of Azua, which were described in the historical chronicles. Some reports suggest that the Azua earthquake produced a tsunami in Ocoa Bay. However, there is no consensus on this issue because two hurricanes caused widespread flooding along the island's southern coasts in August and September 1751. These climatic and seismic events, as well as others that occurred in the 18th century, were reported by Luís Jose Peguero, a creole from Bani, in his book *History of the Santo Domingo Hispaniola Island Conquest, narrated in the year 1762* ^7^.

**Figure S4.** (above) Map of the northeast Caribbean Plate displaying fault zones and microplate boundaries. The inlet depicts the map location in the Caribbean Basin. The red arrow shows the 18-20 mm/yr movement in the N070ºE direction of the Caribbean Plate with respect to the fixed North American Plate (mod. ^8^). The red dashed-line rectangle shows the location of the study area. Historical earthquakes *M_I_* > 7.0 until 1900 A.D. (black circles) and earthquakes *M_w_* > 5 for the instrumental period (beach balls). VI, Virgin Islands; C, Cuba; J; Jamaica. The main neotectonic structures are EPGFZ, Enriquillo-Platain Garden fault zone; OBFZ, Ocoa-Bonao-La Guacara fault zone; SFZ, Septentrional fault zone; BRFZ, Beata Ridge fault zone; and SJPFZ, San Juan-Los Pozos fault zone. (below) Earthquakes of *Mw* > 6.5 in northern Hispaniola (green) and southern Hispaniola were probably linked to the EPGFZ or underthrusting at the Muertos Trench (red) between 1500 and 2021.

**2. Seismic hazard assessment in southern-central Hispaniola**

Probabilistic seismic hazard analysis (PSHA) results in southern-central Hispaniola show the potential ground-shaking intensity for rock-ground site conditions. It is expressed as PGA intervals (values in cm/s^2^) and for a return period of 475 years (i.e., for a probability of exceedance of 10% in 50 years). Two types of modeling were carried out: (a) taking into account the joint contribution of all seismic sources (Fig. S5a) and (b) considering each seismic source separately (Figs. S5b to h).

Considering all seismic sources, the modeled minimum and maximum PGA values are 272 cm/s^2^ and 902 cm/s^2^, respectively. Therefore, the regional seismic hazard values range from intermediate to very high. The obtained PGA zoning defines a triangular zone of high values framed by the traces of the N-BRFZ, O-OBFZ and W-MT segments (Fig. S5a); outside of this zone, the PGA value decreases. The very high value of 798 cm/s^2^ modeled for the Tortuguero Beach site (-70.675°W longitude, 18.435°N latitude) is compatible with a strong ground motion during an earthquake, which triggered the formation of the observed liquefaction structures. Other locations along the Ocoa Bay coast where water-saturated sediments with upward coarse-grained internal structure outcrops might be able to develop an inverse density gradient, forming the liquefaction structures.

Considering models with separate seismic sources, the obtained PGA distributions are logically subparallel to the trace of each main fault zone. However, each of them contributes to the hazard differently. The hazard is high for the N-BRFZ, O-OBFZ and O-MT segments (Figs. S5c, e, g), intermediate for the E-EPGFZ and BAFZ segments (Figs. S5b, h), and low for the SJPFZ and W-NHFZ segments (Figs. S5d, f). A relevant result of the PSHA modeling is that only the N-BRFZ and W-MT segments can produce PGA values higher than 550-600 cm/s^2^, capable of producing the liquefaction structures at Tortuguero Beach, being, therefore, the most probable seismic sources of their generation.

These results are consistent with the breaking down the hazard for all fault zones into hazard curves for each seismogenic source. Source disaggregation at Tortuguero Beach indicates that the O-OBFZ segment has a low contribution, and the contribution of the rest of the seismic sources is negligible (Fig. 10b). For PGA values ​​> 550-600 cm/s^2^, the probability of exceedance is about 12% for the N-BRFZ, 10% for the O-MT and 4.0% for the O-OBFZ, with the remaining seismogenic sources having less than 1.0 %.

**Figure S5.** Result of the probabilistic seismic hazard modeling in the Cordillera Central and Ocoa Bay of southern-central Hispaniola, expressed as iso-PGA (Peak Ground Acceleration) zones (in cm/s^2^) for a return period of 475 years (i.e., an exceedance probability of 10% in 50 years). The traces of the main seismogenic structures are also included. Shaded relief and abbreviations as in Fig. 9. The main neotectonic structures are segments of the main fault zones and are S-OBFZ, Southern Ocoa-Bonao-La Guacara Fault Zone; O-OBFZ, Offshore Ocoa-Bonao-La Guacara Fault Zone; S-JPFZ, Southern San Juan - Pozos Fault Zone; BAFZ, Bahoruco Fault Zone or Barahona Thrust; E-EPGFZ, Eastern Enriquillo-Plantain Garden Fault Zone; N-BRFZ, Northern Beata Ridge Fault Zone; C-BRFZ, Central Beata Ridge Fault Zone; W-MT, Western Muertos Trough; W-DEEP-NHFZ, Western Northen Hispaniola Fault Zone, deep part; O-MT, Muertos Trough, Ocoa Bay segment.

**3. Ethics statement**

The authors discovered the field site at Tortuguero Beach of Ocoa Bay during the 2021 fieldwork campaign. All the personnel involved in the project had the official authorization to perform field activities from the respective geological departments of the Spanish Universities of Oviedo, Alicante, and CSIC-Instituto Geológico y Minero de España and the Servicio Geológico Nacional of the Dominican Republic, which granted authorization to carry out geological surveys. The data collected during the respectful 2021-2023 fieldwork campaigns viewed the liquefaction structures outcrop carefully and left untouched. Marine shell and coral fragments were carefully extracted from the gravel layer for radiocarbon dating. The National Geological Service of the Dominican Republic has approved the export of minerals and samples with a valid export permit for analysis. No endangered or protected species were harmed or affected during the field activities.

**4. References**

1. Flores, C.F., ten Brink, U.S. & Bakun, W.H. Accounts of damage from historical earthquakes in the Northeastern Caribbean to aid in the determination of their location and intensity magnitudes. *Open-File Report 2011-1133*, 237 p. (U.S. Geological Survey, 2012).
2. ten Brink, U. S., Bakun, W. H. & Flores, C. H. Historical perspective on seismic hazard to Hispaniola and the northeast Caribbean region, *J. Geophys. Res. Solid Earth.* **116**, B12318 (2011).
3. ten Brink, U. S., Bakun, W. H., & Flores, C. H. Seismic hazard from the Hispaniola subduction zone: Correction to "Historical perspective on seismic hazard to Hispaniola and the northeast Caribbean region", *J. Geophys. Res. Solid Earth.* **118**(10), 5597-5600 (2013).
4. Bakun, W.H., Flores, C.H. & Uri, S. Significant earthquakes on the Enriquillo fault system, Hispaniola, 1500–2010: Implications for seismic hazard. *Bull. Seismol. Soc. Am.* **102**, 18-30 (2012).
5. Bakun, W. H. Estimating Locations and Magnitudes of Earthquakes in Southern California from Modified Mercalli Intensities. *Bull. Seismol. Soc. Am.* **96** (4A), 1278-1295 (2006).
6. Byrne, D. B., Suarez, G. & McCann, W. R. Muertos Trough subduction—Microplate tectonics in the northern Caribbean? *Nature* **317**(6036), 420-421 (1985).
7. Sevilla Soler, M.R. Santo Domingo Tierra de Frontera (1750–1800). *Publicaciones de la Escuela de Estudios Hispano-Americanos, [Ph.D. thesis]* 502 p. (Sevilla, Universidad de Sevilla,1980).
8. Mann, P., Calais, E., Ruegg, J.C., Demets, C., Jansma, P.E. & Mattioli, G.S. Oblique collision in the northeastern Caribbean from GPS measurements and geological observations. *Tectonics*, **21**(6), 1-23 (2002).
